# The motivations, institutions and organization of university-industry collaborations in the Netherlands

**DOI:** 10.1007/s00191-017-0495-7

**Published:** 2017-05-04

**Authors:** Isabel Maria Bodas Freitas, Bart Verspagen

**Affiliations:** 1 Grenoble Ecole de Management, 12 rue Pierre Sémard-BP, 127, 38000 Grenoble cedex 01, France; 2 UNU-Merit, PO Box 616, 6200 Maastricht, MD The Netherlands

**Keywords:** Incentives, Motivations alignment, Organization, University-industry collaboration, Innovation policy, O31, O38

## Abstract

This study builds on the economics and organization literatures to explore whether and how institutions and organizational structure complement or substitute each other to create specific spaces of alignment where specific individual actors’ motivations co-exist. Focusing on university-industry collaborations, the study examines whether and how different axes of alignment of university and industry motivations are integrated in projects with specific technological objectives and organizational structures, benefitting from the presence of specific institutions designed to facilitate collaboration. Empirically, the study relies on in-depth data on 30 university-industry collaborations in the Netherlands, and provides preliminary evidence that the technological objective and organizational structure of collaboration are malleable variables allowing the integration of both partners’ objectives and expectations. Different institutional incentives for university-industry collaboration favor specific axes of alignment of motivations and certain types of collaborative projects’ design. Hence, our exploratory results suggest that specific organizational and technological structures tend to prevail in the presence of specific institutions.

## Introduction

It is well established in the literature that institutions (Bloom and van Reenen, 2007, Bloom and Van Reenen 2010; Lam 2011), as well as organizational structures (Birkinshaw et al. 2002; Fang et al. 2010), by targeting different individual motivations, create incentives that play a role in economic decision-making about innovation and different types of knowledge activities. But, can organization alone create specific incentives (also to counteract institutional incentives) or are institutional incentives required to be in place for certain knowledge and innovation types of activities being performed? In other words, are organizational and institutional incentives complementary or substitute?

There is abundant evidence showing that specific behaviors, knowledge specializations, and outcomes are prevalent in specific institutional set ups (Nelson 1993; Bloom and Van Reenen 2010), and among groups and organizations with specific organizational structures (Argyres and Silverman 2004; Cassiman et al. 2010; Lazaric and Raybaut 2014; Gambardella et al. 2015). Yet, these contributions have neglected a) the process of motivation alignment, i.e. the fact that certain innovative outcomes may require not only the creation of individual incentives for actions but most importantly that motivations of different actors are reconciled, and b) the fact that both institutions and organizational structures are simultaneously drivers of incentives for specific innovative motivations and behavior. Hence, despite the extensive literature on institutional and organizational incentives, we still do not know if organizational structures can substitute for institutional incentives.

Our paper is an attempt to address this research question by focusing on how institutions and organizational structures provide ‘spaces’ for specific forms of alignment of individual motivations. By motivational alignment we mean integration of the objectives and plans of partners, operating in different technological and institutional environments, into projects and activities that may bring achievement to the parts involved. While motivational alignment appears to be a very theoretical concept, in practice, the setting up of a collaboration depends on the satisfactory alignment of research objectives and expectations of the partners (Foray and Steinmueller 2003; Ankrah et al. 2013). Therefore, we operationalize motivational alignment by the co-existence of the different partners’ motivations for collaboration.

Focus on the alignment of motivations is particularly important in collaborative contexts with partners characterized by diverse incentives. The different incentive frameworks in academia and industry have been blamed for constraining university-industry collaboration and its outcomes (Dasgupta and David 1994; Rosenberg and Nelson 1994). Industry and university have different motivations for collaborating, and these motivations are not additive or equal even within a specific project (Lee 2000; Lam 2011; Ankrah et al. 2013; Subramanian et al. 2013). These differences in research objectives and incentives may lead to or are reflected in organizational differences in values, priorities and time schedules, and may pose further barriers to the co-existence of motivations for university-industry collaboration (Feller et al. 2002). In addition, the presence or absence of institutions to support motivational alignment towards collaboration, such as direct public support for university-industry collaboration, university Technology Transfer Offices (TTOs), part-time professorships, and more active application of university IPR (OECD 2003), may work to reinforce certain motivations for collaboration and/or certain organizational and technological arrangements, or they may crowd them out.

Existing literature has shown that university and industry researchers motivations for collaboration in R&D development are not necessarily in conflict (Lee 2000), and when contradictory they can often be reconciled into a collaborative project with specific technological objective and organization, eventually relying on the presence or absence of different institutions (Lam 2011; Subramanian et al. 2013). Some theoretical taxonomies were developed focusing on the rationale for different university and industry motivations going together, or for conflicting motivations being reconciled (Bonaccorsi and Piccaluga 1994; Arza 2010); and for some knowledge characteristics (tacitness, approppriabilty and universality) going together with a specific degree of formalization and communication (Bonaccorsi and Piccaluga 1994). While expanding existing understanding on motivational alignment for collaboration, these contributions ignore the organizational structures in which these motivations are exercised, and/or to the presence or absence of institutions to support motivational alignment.

To address this gap in our understanding of how organizational structures and institutional incentives compete to influence motivational alignment for university-industry collaboration, our study is guided by a set of interrelated research questions. The first concerns which university and industry motivations can be aligned (i.e., different motivations that work together or can be reconciled towards the outcome), and which motivations create particular trade-offs (i.e., a potential motivation for one party that is difficult to co-exist with a particular motivation of the other party). The second research question refers to how particular sets of motivation alignments are associated with the nature, design/organizational structure and institutional setups of collaborative university-industry projects. Empirically, the present study relies on in-depth data on 30 university-industry collaborative projects in the Netherlands. Our exploratory results suggest that specific organizational and technological formats tend to prevail in the presence of different institutions.

The paper is organized as follow. Section 2 reviews the literature and develops an analytical framework. Section 3 discusses the methodologies used to collect and to analyze data. Section 4 presents the results of the analysis. Section 5 discusses the results. Section 6 concludes and discusses some policy implications of our results.

## Motivations, organizational structures and institutions

Different streams of literature have examined incentives and motivations, using diverse research methods and addressing different research questions. Despite the wide amount of work on incentives and motivations, no overarching framework has been proposed to interpret and to shed light on how different axes of motivation alignment relate to the organizational structures in which these motivations are exercised, and to the presence or absence of institutions drawn to support motivational alignment. This study is set up as an attempt to achieve this ambitious objective. Next, we propose a conceptual framework of the motivational alignment process in collaborative projects with specific technological objectives and organizational design. Our framework builds on and can contribute to the economics, organization and institutional approaches.

In economics, incentives or motivations are at the center of the analysis as they underlie decisions, behavior and performance. The economics of innovation literature has focused on examining the impact of different innovative behavior and choices on performance (i.e. the effect of collaboration on firms’ innovation returns (Belderbos et al. 2004), as well as on the motivations underlying specific desirable behavior and outcomes (i.e. engagement in collaboration for innovation (Tether 2002). However, this literature has not attempted to disentangle and understand the dynamics among the organizational structure of the collaboration and prevalent institutions in which the motivational alignment occurs.

The organization literature has provided extensive evidence on how differences in organizational structures lead to different outcomes and performance, and, in some cases, it has also established causality between specific types of structures and specific types of knowledge objectives (Gulati and Singh 1998; Ranft and Lord 2002; Cummings and Kiesler 2007). However, these contributions have focused on a one-to-one relationship between single dimensions of the organizational format and structure, and the achievement of specific knowledge objective, assuming that the different dimensions of organizational structure are independent among them, and independent of the prevailing institutions.

The institutional approach has well documented that specific behavior, organizational structures and performance may not be dissociated from the environment in which specific social and institutional mechanisms operate (Nelson and Sampat, 2001). For instance, the prevalence of academic patenting and consultancy activities, or the financial performance of TTOs has been shown to depend largely on the characteristics of the universities and of the institutional contexts (Jensen and Thursby 2001; Jensen et al. 2003; Henderson et al. 2005; Nelson 2004; Curi et al. 2015). This approach has, however, mostly neglected the possibility that the organizational design of the collaboration and individual motivations also account for specific patterns of behavior and outcomes (Nelson 1991).

### A conceptual framework

**Diagram 1:**
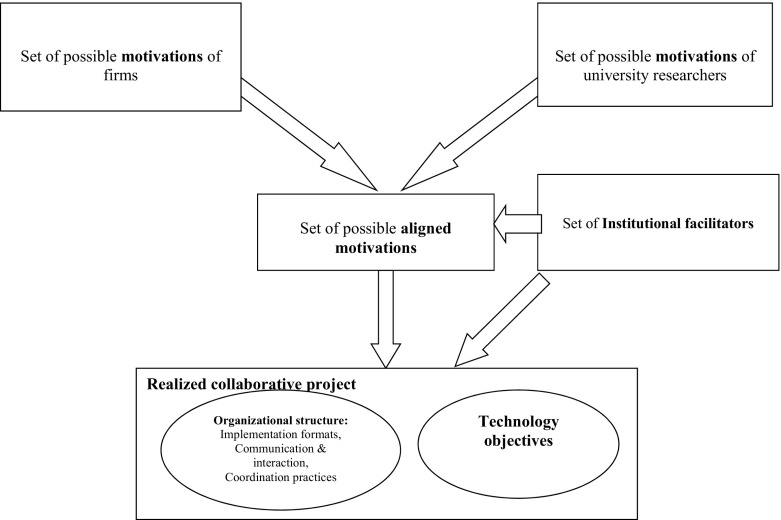
Conceptual framework for analysing university-industry collaborations

In our framework, summarized in Diagram 1, we conceptualize collaborative projects (between universities and private firms) as a space for universities and private firms trying to achieve their technological (or innovation) objectives within a specific organizational format in the presence or the absence of specific institutions. The project is then characterized by a specific technological objective, as well as by specific form of implementation, communication and interaction among the involved researchers (and others), and by specific coordination rules. These factors (the circles in the diagram) are the main features of the design/structure of collaborative projects we measure in this study.

**Fig. 1:**
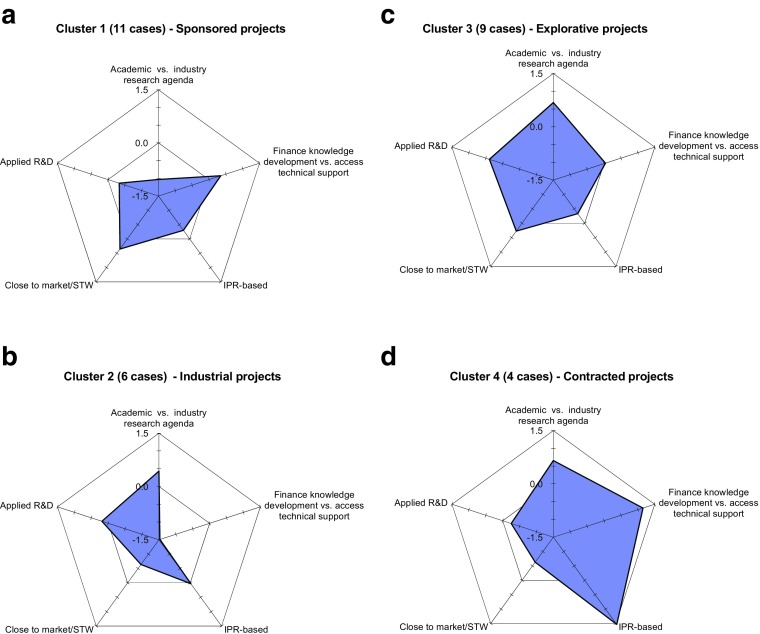
Motivational space of sponsored, industrial, explorative and contracted projects

We identify two main groups of determinant variables that influence how projects are set up, conducted and concluded. One group is considered exogenous, the other, i.e. the set of aligned motivations, is considered endogenous and is modelled below. The exogenous group of factors includes institutional facilitators. Facilitators take place (somehow) within the university and /or private firm, including part-time researchers who work in a private firm and in a university, TTOs, and previously patented knowledge owned by the university. It also includes facilitators, beyond the organizations that are involved in the collaboration. These institutional facilitators emerge from a set of policy instruments used to stimulate university-industry interaction, such as specific subsidy programs for collaborative projects. In the Dutch case, they include programs offered by the STW foundation, which is part of the Dutch research council, aimed specifically at university-industry interaction.

The set of aligned motivations for collaboration among university researchers and private firms depends on several other factors. Alignment stems first from the different (not necessarily conflicting) motivations of the parties in the collaboration, i.e., industry and university. Although in reality these sets of motivations are influenced by such factors as the set of technological opportunities, our case study database does not allow detailed variation of these factors; thus, we consider them as belonging to the exogenous motivations.

By their nature, some of these motivations on either side of the collaboration can be expected to complement each other, e.g., university searching for industrial applications with firm’s product development objectives. This complementarity is expected to be a dominant explanatory factor in the set of aligned motivations, but there may also be trade-offs and conflicts among motivations preventing their co-existence. An example of a trade-off in the alignment process might be the conflict between the university objective of opening up new research avenues and the firm’s product development. Opening up new research avenues would seem aligned to basic research, while product development would involve applied research based on the outcomes from basic research. However, the set of aligned motivations depends also on the institutional facilitators. Next, we discuss the main university and industry motivations for collaboration, as well as the incentives that specific institutional facilitators can create for motivational alignment.

### University and industry motivations for collaboration

Firms collaborate with universities mostly to access and develop interdisciplinary scientific capabilities to solve complex industry problems and to support product development, but also to access public sponsorship. Firms may also collaborate with universities to conduct exploratory, non-targeted research to generate ideas, build technological options and search for new products, technologies and markets, and to get access to skilled labor, especially qualified engineers (Meyer-Krahmer and Schmoch 1998; Lee 1996, 2000; Feller et al. 2002; Carayol 2003; Lam 2005; Balconi and Laboranti 2006; Arza 2010; Subramanian et al. 2013).

University researchers are mostly motivated to collaborate with firms to try out practical applications of their theory and research, and to advance and complement their research agendas Lee 1996, 2000; Perkmann and Walsh 2009; D'Este and Perkmann 2011). On the other hand, they may be motivated by the need to get additional funding and resources to facilitate their research and finance graduate students and purchases of laboratory equipment, as well as to establish a foundation for future research and collaboration opportunities (Lee 2000; Lam 2011).

Ankrah et al. (2013) show that collaborations were set up despite the university and the industry partner differing in their specific motives for collaboration. However, since motivations are not independent or additive, there is not necessarily a one-to-one relationship between the partners’ motivations (Lee 2000; Perkmann and Walsh 2009; Subramanian et al. 2013).

### Institutional facilitators of the alignment of university-industry motivations for collaboration

Different incentives have been introduced to encourage university-industry interaction, collaboration and knowledge transfer, related to collaborative research, setting up of TTOs, part-time professorships, and more active use of university property rights. These incentives may crowd out or reinforce certain university and industry motivations, and may support or substitute collaborative projects with specific organizational structures. Table 1, at the end of this subsection 2.2, summarizes the expectations, which can be drawn from the literature.

**Table 1:** Relationships between institutions, and university and industry motivations for collaboration and organizational structure of the collaboration drawn from the literature

Institutions	University motivations	Industry motivations	Organizational design/structure of the collaboration	
			technological objective	Organization/ Implementation
Public research sponsoring	Access research funds to complement research activities; Build research network for future research;	Access public funds to co-fund research/product development	Knowledge related to existing technologies	(?)
Part-time professorship	Access research funds; Build research network;	Access public funds to co-fund research/product development	Knowledge related to existing technologies	(?)
Academic patents	Access research funds; Get insights into the applicability of previous research results	Explore R&D opportunities for new product development	Knowledge related technologies substituting for existing ones	(?)
TTOs	(?)	(?)	(?)	Consultancy or licensing contracts

(?) indicates lack of empirical support from prior literature

#### Grants for collaborative research

Public grants may create incentives for specific motivations for collaboration and for specific alignment processes. Participation on public sponsored collaborations seems motivated by access to complementary knowledge and resources; hence often it is not critical to firms’ competitive position, but it provides a collaborative space where opportunistic behavior is not perceived as a severe problem (Tripsas et al. 1995; Sakakibara 1997). For example, focusing on inter-firm collaboration, Matt et al. (2012) show that EU sponsored collaborations concern more peripheral and exploratory activities, and involve less conflicts, higher administrative burden and less intense interactions with partners than non-sponsored collaborations.

In the case of university-industry collaboration, public grants were shown to provide incentives for trust building among the partners, and to influence the extent of development of new products or processes that are the direct outcomes from collaboration (Okamuro and Nishimura 2015), and the ability to gain knowledge and rent spillovers (Nishimura and Okamuro 2016). Hence, public sponsored university-industry collaboration is expected to permit the co-existence of university motivations to access research funds to complement their research activities and to maintain/build their research networks for future collaboration (Lee 1996, 2000; Perkmann and Walsh 2009; D'Este and Perkmann 2011), and of industry motivations to complement and co-fund their research agenda (Balconi and Laboranti 2006).

#### Part-time professorships

In some countries, part-time Chairs have been institutionalized with specific regulations as an incentive for university-industry interaction and collaborative forms of knowledge transfer. It has been suggested that exchanges between industry and university, which enable wider social and industrial networks and improve market awareness, make researchers more productive in developing innovations for industry (Dietz and Bozeman 2002). In particular, Zucker et al. (2002) show that employment of top university researchers by entrepreneurial start-ups positively influences the success of these spin offs.

When an industry researcher splits his time between university and industry, his industry research objectives will dominate (Lam 2011). Hence, collaborative projects involving part-time professors can be expected to reflect an alignment among the industry motivations to access public funds to support an industry R&D agenda and the university motivations to obtain additional funding for university research and to increase networking opportunities. The objective of increasing knowledge related to existing technologies may then be more frequent in collaborative projects that involve part-time professors, especially if these professors are paid by the firm.

#### University property rights

OECD country governments have been defining and revising regulations related to the rights on university research results with the aim of facilitating knowledge transfer between university and industry (OECD 2003). Several studies examine the impact of regulation on university patenting, on the effectiveness of university-industry knowledge transfer, especially in the US context (e.g. Lee and Gaertner 1994; Jensen and Thursby 2001; Feller et al. 2002; Jensen et al. 2003; Henderson et al. 2005; Mowery et al. 2015). Based on detailed case studies, Colyvas et al. (2002) argue that university patents seem important to induce firms to develop ‘embryonic’ inventions, but not to adopt almost ‘ready-to-use’ innovations. Lee and Gaertner (1994) argue that encouraging universities to focus on industry value added, and especially patenting and licensing, may not ensure that firms will develop commercial products sequentially. We may then expect that more active university patenting might encourage collaborations that focus on knowledge developments related to technologies substituting for existing ones that are likely to be commercialized. Thus, in projects involving academic patents, industry may be expected to collaborate with the shorter or longer term objective to explore R&D opportunities for new product development, and university may cooperate to expand research funds or/and to get insights into the applicability of previous research results.

#### University TTOs

OECD country governments also encourage the creation of university TTOs (OECD 2003). TTOs were conceived to facilitate technology transfer to industry and commercialize university knowledge. The growth in university TTOs has been associated with clarification of university patenting rights as well as campaigning for university entrepreneurship. Although some TTOs have been successful in increasing university funding (though not always sufficient to cover the TTO’s running costs), the additionality of TTOs in terms of effective technology transfer to firms is difficult to prove (Bozeman 1994; Colyvas et al. 2002; Bach and Llerena 2007). TTOs are a service infrastructure within the university the role of which is not so much to encourage collaboration, but to support university researchers with the administration of licensing, consultancy contracts, patenting and spin off creation (Curi et al. 2015). Hence, TTOs are expected to have a limited role in influencing the process of motivational alignment if not in the presence of specific implementation forms, such as consultancy or licensing contracts, for which the university may have created specific templates.

Besides institutional facilitators, specific organizational design and technological objectives may permit accommodating the university and industry objectives. Next, we review the literature on how the different organizational structures may facilitate the co-existence of specific motivations for collaboration.

### Organizational structure of collaboration as a space for alignment of motivations

Accommodating the specific objectives and motivations of both collaboration partners may require some balancing among the dimensions of the organizational structure and the technological objectives of the joint project. The technological objective and the organizational structure of collaborations, defining the cognitive objectives, incentives and coordination rules, are variables that the partners must integrate with their motivations, expectations, and concerns to benefit from the collaboration (Gulati and Singh 1998; Avadikyan et al., 2001, Chompalov et al. 2002; Ranft and Lord 2002; Foray and Steinmueller 2003; Sampson 2007). In other words, the technological objectives and the organizational structure of projects may counter differences in the incentive structures of the partners and create a space for the alignment of their motivations and objectives. At the end of this subsection 2.3, Table 2 summarizes the expectations that can be drawn from the literature.

**Table 2:** Relationships between organizational structure of the collaboration and university and industry motivations for collaboration drawn from the literature

Organizational design/structure of the collaboration		University motivations	Industry motivations
Technological objective	Organization/ Implementation		
Knowledge related to existing technologies	(?)	Build research network; Access research funds; Applicability of previous research results	New product development
Knowledge related technologies substituting for existing ones	(?)	Applicability of previous research results	Explore R&D opportunities for new product development
(?)	PhD or Master thesis	A variety of motivations	A variety of motivations
(?)	Licensing or consultancy contracts	Access research funds; Applicability of previous research results;	New product development
(?)	Intensive and informal communication	Applicability of previous research results	New product development; Solving technological problems
(?)	Less intensive communication	(?)	Access new knowledge, Explore R&D opportunities for new product development
(?)	Technical difficulties	Applicability of previous research results y	(?)
Knowledge related technologies substituting for existing ones	Market problems	(?)	New Product development
(?)	Cultural differences	Access research funds; Build research network	New product development

(?) indicates lack of empirical support from prior literature

The technological objectives of the collaboration mirror the early phase of the alignment process among both partners, i.e. the integration of their diverse interests and motivations into a common knowledge development objective. The organizational or implementation structure of the collaborative project mirrors the later phase of the alignment process on ‘how’ to implement the agreed ‘what’. More precisely, the organizational structure of the collaborative project (the implementation form, the forms of communication and interaction, and coordination problems) reflects an agreed division of labor, its respective organization and coordination of the knowledge production and distribution, and agreed rules for accessing resources during development, and appropriating and diffusing project outcomes (Foray and Steinmueller 2003).

#### Technology objectives

The literature emphasizes the importance of knowledge characteristics such as level of codification, appropriability, and universality in shaping the knowledge development and transfer processes (Avadikyan et al. 2001; Birkinshaw et al. 2002; Zhang et al. 2007). In a collaborative project, these characteristics are to a large extent associated with the degree to which the technology is in an early development stage versus in a near-to-market phase and to the degree of proximity to the partners’ core knowledge (Cowan et al. 2000; Hoetker and Mellewigt 2009; Gambardella et al. 2015). Another general knowledge characteristic often considered in the innovation studies literature and loosely associated with those dimensions is the degree of originality or novelty of the project objectives (Cassiman et al. 2010).

In line with the literature, collaboration for the development of near-to-market knowledge, involving a higher degree of applicability, codification and specificity, but also posing more problems of appropriation (Shenkar and Li 1999; Ancori et al. 2000, Metcalfe 2005) is expected to be the outcome of integration of industry motivations related to new product development with university motivations to develop possibilities for future collaboration, and obtain research funding or/and explore the applicability of previous research. Collaboration for projects focused on novel technologies, especially on technologies that are substitutes for existing ones, may require instead that university is motivated to explore applicability, and industry to explore new knowledge development.

#### Organizational or implementation structure of collaborative projects

##### Implementation form

The implementation of knowledge development and knowledge transfer is often dictated by the length and type of the relationship (Hall et al. 2001; Chompalov et al. 2002; Cummings and Kiesler 2007). Implementation formats are specific to the type of interaction examined, i.e. knowledge acquisitions, knowledge transfer within the same organization, collaboration, and so on. However, very few studies examine these dimensions empirically, especially the formalization of the relationship, and its combination with other organizational dimensions.

University-industry collaboration can be implemented through involvement of students, consultancy and licensing contracts with university researchers, and/or spin offs. Student involvement refers to continuous research for a longer (PhD) or shorter (Master) period of time, which is supervised by a professor who provides informal advice on the developments in the firm related to the project. Being the main forms of implementation of university-industry collaboration, these forms may permit a variety of alignment spaces (Salimi et al. 2015). Licensing and consultancy contracts involve more specific and formal agreements about tasks and duration of the collaboration, and procedures to resolve conflicts, e.g. over IPR (Mowery et al. 2015). These implementation forms are expected to permit integration of industry motivations for new product development with university applicability or expand research funds motivations.

##### Forms of communication and interaction

The characteristics of communication reflect the agreed arrangement for labor and knowledge division in the project. Frequency and type of communication are among the most frequent characteristics examined in relation to knowledge development, transfer and acquisition (Gupta and Govindarajan 1991; Bonaccorsi and Piccaluga 1994; Chompalov et al. 2002; Cummings and Kiesler 2007; Lazaric and Raybaut 2014). Therefore, we consider both the frequency and the (in)formality of the communication during the collaboration.

Projects in which industry is motivated for getting support for product development or solving technological problems as opposed to accessing new knowledge, getting new ideas and building options, are expected to require more intensive and diverse communication. They may be aimed at developing universal knowledge, but are more likely set up to find solutions to specific industry problems. Intensive and open communication seems to be important for the development of solutions to industry (Mohr and Spekman 1994; Lazaric and Raybaut 2014). Similarly, in projects where the university motivation is to test the applicability of its research results, communication is expected to be intense and informal, especially when it is impossible for the industry partner to conduct parallel research on its own (Mohr and Spekman 1994; Osterloh and Frey 2000; Perkmann and Walsh 2009).

##### Coordination practices

In addition to accommodating different motivations, the organizational structure of the collaboration sets implicit or explicit the coordination rules (Osterloh and Frey 2000; Ranft and Lord 2002; Sampson 2007; Ornston and Schulze-Cleven 2015). University-industry R&D collaborations are likely to suffer from technological and market problems, and conflicts emerging from differences in the cultures of the two parties, their objectives, and interest in appropriability and commercialization (Kline and Rosenberg 1986). These problems in part reflect the (in)efficiency of the coordination practices (formal or informal) in place, and can lead to the project being abandoned.

Technical difficulties related to knowledge development and industry exploitation of scientific knowledge (e.g., scaling up university samples, applying knowledge to specific materials, developing a user-friendly product) are expected to be more frequent in projects where the university’s objective is to test the applicability of its research results; thus, when the knowledge objectives relate to technologies to substitutes for existing ones. Market problems, such as lack of customers, change to the industry partner’s marketing strategy, or competition (a ‘technology race’) may in turn be more frequent in projects that focus on developing near-to-market knowledge. Conflicts emerging from different attitudes to knowledge development and sharing, appropriability and applicability are expected to be more likely in projects where the firm is motivated by objectives of product development exploiting university research and the university researchers are collaborating to find funding or on the expectation of collaboration and funding opportunities in the future.

## Methods

### Data

Although the conceptual framework suggests a number of causal linkages, it is not the aim of our empirical analysis to test these causal relations. This is left to future research. Our objective here is to use the framework to guide our thinking about how the various aspects of the collaboration and motivational alignment are related to each other, and to interpret the relationships observed in the cases in our database. In this manner, we hope to advance existing knowledge on the complementarity between organization and institutions in supporting certain axes of motivational alignment.

Analysis and development of unidentified relationships and concepts, such as our objective of examining the association among motivational alignment, organization and institutions, require a research design that provides in-depth information on multiple cases (Eisenhardt 1989). Therefore, we collected in-depth information on 30 university-industry collaborations, in the Netherlands. The university partners we study are all Dutch universities and most (but not all) firms are Dutch companies.

Our unit of analysis is the piece of knowledge developed or co-developed by the university and transferred to one firm or a group of industrial firms, independent of whether or not it is absorbed, used or commercialized. We underline that our collaborative projects were selected on the basis of knowledge development and eventual transfer, not on the basis of the participating firm having recognized the value of the knowledge and having decided to use and (subsequently) commercialize it.^1^ We focus on projects that have been concluded in order to collect information on the project’s organizational structure from origin to conclusion including achieved outcomes. This focus on completed collaborative projects may bias our sample to successful cases. However, project performance (apart from completion) was not a factor in case selection; indeed, our sample shows great variety of project performance.^2^ Hence, we are confident that potential selection bias does not pose serious problems for our research design.

We used a mix of strategies to identify the cases. We searched national electronic libraries for PhD theses completed in the previous five years that acknowledged support from industry and research grants from national research councils. We interviewed the chairs of some research departments in the faculties of mechanical engineering, biotechnology, chemistry, applied physics and electrical engineering, in two technical universities in the Netherlands (Eindhoven University of Technology and Delft University of Technology), and the directors of these universities’ TTOs. We identified professors with large numbers of industrial patents choosing two for our sample.

Multiple in-depth case research design requires a different logic to sampling; it is important to select a variety of examples cases of the phenomenon of interest to allow comparison and contrast (Eisenhardt 1989; Yin 2009). Hence, the 30 collaborations were chosen to ensure variety on four axes. First, collaborations should have diverse disciplinary origins. Second, they should show variety of forms of financing and design (we chose cases financed by the university, the STW,^3^ other research sponsors, and firms). Third, collaborations must show diversity among the efforts of the university and the firm in relation to the origins and development of the innovation (university-driven research; the firm addresses the university with the idea; results from collaborative project). Fourth, we sought variety between formal and informal forms of knowledge transfer and university-industry interaction (i.e. a few cases of creation of start-ups or spin offs and university patenting). Fifth, there should not be more than one project with the same university and company. Table 3 provides information on the variety in the sample. The University of Eindhoven provided 19 cases, Delft University eight and the University of Leiden three. Eindhoven and Delft are technical universities including mainly applied disciplinary faculties, while Leiden is a classical university.^4^

**Table 3:** Some information on the cases of collaborative projects chosen

		N. Cases
Disciplinary	Biomechanics	2
	Biology/ Medicine	3
	Chemical/ Materials	3
	Applied Physics	8
	Electrical engineering	7
	Mechanical engineering	7
Origin of project attributed to	University	13
	Firm	11
	Previous / On-going collaboration	11
	Involving part-time professors	7
	Involving former industrial researchers	6
	Based on previous patents	13
	of which univ. patented knowledge	5 (3 university owned)
Finance of R&D collaborative project	Research sponsoring (STW)	16 (8)
	of which without other sources	3
	Firm	9
	University	2
Outputs	Patents output	16
	Spin offs	7

Data collection was done in a retrospective way and involved a combination of primary data (interviews) and secondary data (such as doctoral theses, public data from research sponsors, public information made available by the collaborating partners, patent databases, and so on as applicable) (Van de Ven and Poole 1990). Data were collected on the technological and organizational aspects of the collaboration from its origins to the end of the research project (Kingsley et al. 1996; Bozeman 2000; Avadikyan et al. 2001, Cummings and Kiesler 2007), including the characteristics of the innovation/invention developed, the origin and format used for project implementation, the roles of the partners, form and frequency of interactions, difficulties and conflicts/power imbalances, and participation of supporting institutions. We also collected information on the type of knowledge and innovation developed in the project and some characteristics of the actors involved.

Primary data on each collaboration was collected in between two (only in one case) and six semi-structured interviews with researchers in the firms and universities involved in the projects (three is the median).^5^ Interviews were recorded, which facilitated retrieval of information. A four to six page summary of the collaborative development process of the focal piece of knowledge has written after the interviews. In addition, we developed a standardized protocol that permitted systematization of the information collected, and insured we had information about all the steps of the collaboration for every case which could enable codification and comparison of across the different cases. The protocol includes over 200 questions requiring short written answers, and was filled by MSc engineering students after the interviews. The summary was discussed by the data collectors and the coordinator of the data collection (author of this study), the different steps and aspects of the collaboration were scrutinized, the filled protocol examined, and in almost all the cases, another contact with the interviewees was done to collect additional information. Codification of the cases was done by the data collector and by data collection coordinator. In the few cases of discrepancy, the coders discussed the nature of the situation that was being coded and the content of the codes. It was often decided to contact again the interviewees to get a better understanding of the situation. In the few cases of conflict, the specific case was further scrutinized and the codes were discussed until clarification and unanimity in the decision.

We converted the information derived from the responses to the protocol questions into binary variables (Van de Ven and Poole 1990; Chompalov et al. 2002; Carayol 2003). For the purposes of this study, we used only the information on the university and industry original motivations for collaboration, design of collaboration (including technological objectives and organizational structure), and presence of institutional incentives.

After a dialectic process between the early codified data and the literature, we retained four university and four industry motivations.^6^ Four dichotomous variables capture information on the main university motivations to collaborate with industry (*Applicability*; *Future research opportunities*; *Financing research*; and *Maintain collaborative contacts*), and four other dichotomous variables refer to the industry motivations to collaborate with university (*Product development*; *Technological problem*; *Access to public funding*; *Research opportunity*).

Eleven variables characterize the design of the collaborative projects. Specifically, two variables, *Substitute technology* and *Commercialization*, characterize the originality and the development stage of the technological objectives of the project, dimensions commonly used to characterize knowledge development activities and projects (Cassiman et al. 2010). As the benefits and outcomes of the collaboration are closely coupled with the motivations and objectives (Lee 2000, Hall et al. 2001), we used information on the project knowledge objectives and its outcomes to characterize the project’s knowledge development objectives. Knowledge that substitutes existing technologies, in theory, has a lower possibility of codification and specificity and eventually higher degree of originality than knowledge related to new technologies that complement existing ones. Commercializable knowledge, in theory, has a higher degree of applicability, codification and specificity, but may pose more problems of appropriation (Shenkar and Li 1999; Ancori et al. 2000, Metcalfe 2005). Hence, projects in which knowledge outputs are commercialized or in process of being might have focused on near-to-market knowledge development efforts.

The other nine variables characterize the organizational structure of the collaborative project. Two variables characterize the frequency and the informality of communication and interaction between the parts during the project development, most common studied dimensions in knowledge transfer activities (Mohr and Spekman 1994; Ranft and Lord 2002). *Frequency* is a dichotomous variable that provides information on whether communication and interaction occurred often versus occasionally. *Informal* is an ordinal variable capturing the level of informality; it takes the value 0 if interactions and communication often had an only a formal nature, the value 1 if some informality was observed, and the value 2 if there were a high informality in communication and interaction among the parts. Four dichotomous variables capture information on the most common modes of university-industry forms of implementing collaboration: *PhD thesis*; *Master*, *licensing/consultancy* contracts and *spin offs* (D’Este and Patel, 2007; Bekkers and Bodas Freitas, 2008). Three other dichotomous variables capture information on the occurrence or not of coordination problems during the project caused by *technological problems*, *market dynamics* and *cultural differences*.

Finally, four dichotomous variables provide information on whether the project occurred in the presence or absence of the following institutions: *Part-time research position, public research funding for collaborative research projects, STW research funding, and university TTO*. In order to take into consideration that little university knowledge is patented by the university, the variable *Previous university patented knowledge* captures information on whether university-developed knowledge has been patented and is owned either by the university or a private firm.

Table 4 provides detailed information on each variable used in this study to explore the alignment axes of university-industry motivations and their association with specific organizational characteristics of the project and how certain institutions encourage or prevent the alignment of university-industry motivations.

**Table 4:** Variables used in the analysis

Variable	Description
Motivations	
Variable group University motivations	
Applicability	The university researcher is interested in developing industrial applications of previously developed (basic) knowledge
Future research opportunities	The university researcher is interested in obtaining knowledge that will open up new research avenues
Financing research	The university researcher is interested accessing additional funding for undertaking research
Maintain collaborative contacts	The university researcher is interested in building and maintaining a network of industrial contacts that will enhance her reputation and performance as a researcher
Variable group Industrial motivations	
Product development	Industry is interested in developing specific new products or services
Technological problem	Industry wants to solve a specific technological problem encountered in commercial practice
Access to public sponsoring	Industry is interested in obtaining additional funding to achieve its R&D agenda.
Research opportunity	Industry is interested in exploring technologies that are judged to have future commercial potential
Design of collaboraiIve project	
Variable group Technological objectives	
Substitute technology	The technological goal and outcome substitutes for an existing technology (used by the firm or by others in its markets)
Commercialization	The project led to a technology that has actually been commercialized or is in the process of being commercialized
Variable group Project implementation form	
PhD thesis	An important part of the project led to a PhD thesis
Master	An important part of the project led to an MSc thesis
Licensing/consultancy	The project involved a licensing and/or consultancy contract
Spin off	The project involved or led to the creation of a spin-off company that employs university researchers
Variable group Communication and interaction during the project	
Frequency	Interactions occurred often versus occasionally
Informal	Interactions often had an only a formal nature(0) some informality (1), high informality (2)
Variable group Coordination practices and problems during the collaboration	
Market dynamics	Developments in the market in which the industrial participant operated affected the project in a negative way (e.g., lack of customers or strong competition)
Technical problems	The project encountered severe technical problems in implementing technological principles
Cultural differences	The project suffered from a misalignment of the cultures in university and industry
Institutions	
Variable group Institutional facilitators	
Part-time researchers	The project was facilitated by the participation of researchers with a part-time appointment in industry and part-time appointment in university
Univ. TTO	The university TTO was significantly involved in initiating and/or managing the project
Previous univ. patented knowledge	The project involved the application of university-developed knowledge that was previously patented (owned by the university or the firm)
Research Sponsoring	The project received funds from a third party
STW	The project was carried out as part of a programme of the Technology Foundation STW, and was funded by STW

### Data analysis

We use a statistical methodology to analyze the data collected on the 30 collaborations. Compared to pure qualitative methods, we think that our choice has the following advantages. First, our protocol is standardized, which allows us to make meaningful comparisons across cases. Second, our sample is much larger than the norm in case-study based research. Although there may be too few cases to allow strong generalizations, these are sufficient for quantitative analysis. Third, while qualitative analytical methods provide a good basis for the examination of rich and complex processes, involving either uni-dimensional or independent categories, multivariate techniques are better suited to analyzing relationships among non-additive and multi-dimensional categories, such as “motivations”, and “institutions” and “organizational structure”, i.e., the categories in our study. Thus, the generalizations enabled by quantitative methods provide accurate evidence on the axes of alignment of university and industrial motivations and on how they are integrated in projects with specific organizational structures.

We address our research questions in two consecutive steps. Section 4.1 presents the factor analysis used to identify the alignment axes of university and firm motivations in projects with specific organizational and technological characteristics, and the role of institutions in facilitating the different alignment and collaboration formats. The factor analysis uses a *polychoric* correlation matrix to calculate the factor, better to account for the fact that our variables are binary. It addresses the research questions related to whether aligned motivations represent trade-offs or complementarities, and how institutional, organization and policy factors interact with aligned motivations.

The second step in our analysis, in Section 4.2, is to visualize and understand differences in the motivations for and design of university-industry projects and the presence of institutions. We are not interested in the hierarchy of clusters or in proximity to specific cases in our data nor do we depart from a well-known typology of projects. Our objective is to identify prototypes of collaborative projects; thus *K*-means cluster analysis seems the most adequate method. We choose four clusters because that solution maximized the number of variables that according to the ANOVA test are significantly different across clusters. Finally, we provide a summary of an example of a typical project in each cluster. This addresses our third research question (seeking stylized patterns of aligned motivations, organizational and institutional designs and policy).

## Motivational alignment and a typology of collaborative projects

### Axes of university-industry alignment

In all 30 collaborations, the university researchers were motivated to collaborate to engage in high-quality scientific research and develop knowledge. Specific reasons included (in order of frequency): (i) obtaining insights into the industrial applicability of previous research; (ii) maintaining contact with industry; (iii) accessing additional funding; and (iv) increasing opportunities for future (collaborative) research. The motivations for collaboration were not exclusive (the sum of the frequencies of these motivations adds to more than 30); the motivation to maintain collaboration contacts contrasts with the other three motivations.

We identified four main motivations for firms to propose or engage in collaboration with universities. These are ranked in order of importance: (i) support for product development; (ii) access to public research funding; (iii) solutions to technological problems; and (iv) finding research opportunities. As with university motivations, industry motivations for collaboration are not exclusive. The motivation of accessing a research opportunity contrasts with motivations related to solution to technological problems and support for product development projects.

We address our first research question by exploring how alignment of university and industry motivations for collaboration relates to specific project design in terms of its technological objective and organizational structure, and to specific institutional facilitators. We use factor analysis to identify the axes of alignment of university-industry motivations and their institutional and organizational context. In addition to the variables for university and industry researchers’ motivations for collaboration, we include the eleven variables described in Section 3 for the key dimensions of the organizational format and technological objective of the collaboration, and the five variables for the key institutional facilitators. See Table 4. We selected five factors which together explain around 70% of the observed variance. Each factor has eigenvalues greater than two and explains more than 7% of variance (i.e. the factors chosen more than complying with the minimum requirements). The reported factor loadings in Table 5 are rotated using the oblique method, which accounts for correlation among the factors in case it is present; otherwise it provides an almost orthogonal solution. We focus on factor loadings with absolute values >0.4 (Hair et al. 2006).

**Table 5:** Factor loadings of university and industrial motivations, technological and organizational structure of the collaborations, and institutional facilitators of university-industry collaboration

		1	2	3	4	5
Industrial Motivations	Product development	−0.48	**−0.51**	0.29	−0.17	−0.39
	Technological problem	−0.20	**−0.83**	0.25	0.26	−0.15
	Access to public sponsoring	**−0.85**	−0.02	−0.07	−0.13	−0.26
	Research opportunity	−0.07	**0.60**	0.17	0.16	**0.91**
University Motivations	Applicability	**0.59**	−0.21	0.17	0.24	**0.51**
	Future research opportunities	0.11	−0.08	**0.63**	0.01	0.01
	Financing research	0.07	**0.48**	0.35	0.11	−0.08
	Maintain collab. Contacts	**−0.89**	**−0.80**	0.34	−0.02	−0.03
Technological objective	Substitute techn.	**0.89**	0.07	−0.25	0.21	−0.05
	Commercialization	0.14	0.02	0.19	**0.89**	0.26
Implementation form	PhD thesis	0.07	**0.66**	−0.16	**0.59**	−0.14
	Master	0.34	**−0.61**	−0.07	0.06	−0.11
	Licensing/consultancy	0.00	−0.15	**0.91**	**0.44**	0.20
	Spin off	**0.71**	**0.51**	−0.19	**0.82**	−0.03
Communication and Interaction	Frequency	0.38	−0.37	0.24	−0.20	−0.15
	Informal	0.36	**−0.56**	−0.06	0.10	0.23
Coordination practices & problems	Market dynamics	−0.16	−0.16	0.05	−0.32	**−0.88**
	Technical problems	**0.87**	0.27	0.03	0.10	0.04
	Cultural differences	0.18	0.30	**0.44**	−0.07	−0.20
Institutional Facilitators	Part-time researchers	**−0.57**	−0.32	0.15	0.33	−0.01
	Univ. TTO	0.37	-0.08	−0.18	−0.19	0.01
	Previous univ. patented knowledge	0.00	−0.15	**0.91**	**0.44**	0.20
	Research Sponsoring	**−0.53**	**0.46**	−0.21	0.12	0.05
	STW	**-0.60**	−0.05	0.13	**0.50**	0.20
	Cronbach’ Alpha	**0.83**	**0.73**	**0.82**	**0.47**	**0.49**
	% var	24%	19%	11%	9%	7%
	Cum	24%	43%	54%	63%	70%
	Eigenvalues	7.17	5.65	3.39	2.69	2.05

1: 30 Observations

2: Extraction Method- PCA; Rotation Method – Oblique

3: factor loadings with absolute value > = 0.4 in bold

The first factor refers to the two extremes of the axis of alignment of motivations: applicability of university research (universities, strongly positive), and accessing public research funding (industry) and maintaining collaborative contacts (universities) (the latter two strongly negative). The opposite signs of the loadings of these variables suggest a conflict between these sets of objectives, thus creating the academic goals vs. advance industry research agenda trade off axis. Technical problems are a common barrier *(academic goal*), and substitute innovations are often the outcome; spin-offs are relatively frequent. Part-time researchers, research sponsoring and STW are infrequent institutional facilitators for the axis *academic goal*, but dominate on the axis of *advance industry research agenda*.

The second factor also maps two opposite motivational alignment extremes. In this case, the primary conflict is, on the one hand, between motivations in the business domain, i.e., research opportunities recognized by firms, and university funding opportunities (strongly positive loading), and, on the other hand, industry product development and solution to technological problems and maintaining collaborative contacts (universities) (negative loadings). This second factor seems to point to trade off finance knowledge development (i.e. exploring research opportunities) vs. access technical support (i.e. developing products and solving concrete technical problems). At the *finance knowledge development* end, we find a rather formal mode of knowledge transfer (strongly negative loading on *informal*). Projects are often in the form of PhD (but not MSc) research, are sponsored by public research grants, and can lead to spin-offs. The *access technical support* end of the axis involves Masters projects and informal modes of interaction.

The third factor refers to IPRs on university research as an institutional facilitator, and licensing and/or consultancy contracts. We label this the IPR axis. The main motivational factor associated with this axis is universities exploring future research opportunities. Universities use IPR to maintain access to a research field and to resources. Cultural differences are important here. There is minor industry product development motivation on this axis.

The fourth factor characterized by STW as an institutional facilitator, high levels of commercialization, and PhD research projects and spin-offs (no very positive or very negative loadings of any industry and university motivation). This factor also loads high and positive on previously existing IPRs and licensing/consultancy, which is in line with the strong emphasis of STW on IPRs. We label this close to market/STW axis, referring to the fact that projects supported by STW are aimed at commercialization and user involvement.

The last factor shows industry and university motivations aligned in the same direction (i.e., both have positive loadings). The two motivations are research opportunities (industry) and applicability (university). These are applied R&D projects, which are characterized by an absence of market-related barriers. We label this the applied research axis.

### Typology of university-industry projects

#### Identifying a typology of university-industry projects

These principal components can be likened to the dishes on a menu of actual collaborative projects, and are used to address our third research question (about the typology of collaborative projects). None of the 30 collaborative projects is described adequately by only one of the five factors, which should be considered analytical tools used to identify the individual processes involved rather than types of cases. To visualize and understand the differences in objectives, motivations and design of university-industry projects that characterize our collaborations, we propose the following typology. K-means cluster analysis is our main analytical tool, and uses data from the 24 variables used in the factor analysis. Table 6 provides information on the truncated mean for each variable within each cluster. Figures 1a-d present a summary profile of the clusters in relation to the five principal components identified in section 4.1. The figures were constructed based on calculation of average factor scores in a cluster, plotted on a radar plot. Factor scores were calculated using the Bartlett method, and then standardized. A positive (negative) value indicates a higher (lower) than average score, relative to the 30 sample cases.

**Table 6:** Results of cluster analysis

		Sponsored	Industrial	Explorative	Contracted
Industrial Motivations	Product development	1	1	0	1
	Technological problem	0	1	0	0
	Access to public sponsoring	1	0	0	0
	Research opportunity	0	0	0	1
University Motivations	Applicability	0	1	1	1
	Future research opportunities	0	1	0	1
	Financing research topic	0	0	0	1
	Maintain collab. Contacts	1	1	0	0
Technological objectives	Substitute techn	0	1	1	1
	Commercialization	1	0	1	1
Implementation form	PhD thesis	1	0	1	1
	Master	0	1	0	0
	Licensing/consultancy	0	0	0	1
	Spin off	0	0	0	0
Communication and Interaction t	Frequency	0	1	1	1
	Informal	0	1	1	0
Coordination practices & problems	Market dynamics	0	0	0	0
	Technical problems	0	0	1	1
	Cultural differences	0	0	0	1
Institutional Facilitators	Part-time researchers	0	0	0	0
	Univ. TTO	0	0	0	0
	Previous univ. patented knowledge	0	0	0	1
	Research Sponsoring	1	0	0	0
	STW	1	0	0	0
	Number of cases	11	6	9	4

30 cases. The five variables *part-time professor*, *market-related problems*, *TTOs, spin-offs,* and *product commercialised or in process of being commercialised* do not differ significantly across the four groups of projects

We refer to the first cluster (Cluster 1), which contains the largest number of cases as *Sponsored Projects*; all 11 cases in this cluster received third party research funding. This cluster includes six of the eight cases of STW funding. In relation to the motivational space represented in the PCA, this cluster is clearly focused on *advance industry research agenda* and is *close to market/STW*. As in Fig. 1a, average factor scores on *close to market/STW* are positive, but strongly negative for *academic goal*. The main motivation for university researchers to participate in projects in this cluster is to maintain collaborative contacts (eight out 11 cases). Ten of the 11 cases of industry motivation to participate in accessing research funding are in this cluster. Also, in seven of the cases in this cluster, industry is motivated for product development. Thus, this cluster seems to capture cases where funding and maintain collaborative contacts is the motivation for industry and university participation, respectively. These projects tend to be implemented in the form of PhD research, and their outcomes are likely to be commercialized.

The nine projects in the second largest cluster (Cluster 3) are characterized by university researchers being motivated by searching for applications for their basic research (observed in all the nine cases); we call it *Explorative projects*. None of the firm’s motivations in this cluster are strong, but the results of collaborative projects tend to be commercialized. In terms of the principal component axes, as plotted in Fig. 1c, this cluster has broad support in *academic goals* (rather than *advance industry esearch agenda*), *applied R&D* and *close to market/STW*. This cluster is characterized also by high frequency of technological problems (7 of the 9 cases). These projects tend to focus on technologies that substitute for existing ones, to be implemented by PhD students, involve high frequency and informality of communication between the partners, and outcomes are likely to be commercialized.

The clusters 2 and 4 are smaller (cluster 1 and 3 account for 20 out of the 30 cases). However, since our sample of cases does not pretend to be representative, cluster size is not an indication of importance. Cluster 2 has six cases, and includes a relatively large variety of university motivations. The most observed industry motivations are accessing support for product development and developing solutions for technological problems. In terms of the principal component axes, as in Fig. 1b, it has a base in the *access technical support, academic goal* and *applied R&D* principal components. These projects are usually implemented by Masters students and focus on researching and developing proof of concepts and prototypes of substitutes to existing technologies, in an environment of intense formal and informal university-industry interaction. We label this cluster *Industrial projects* because they are aimed at solving technological problems in industry product development projects*.*

Cluster 4 (four cases) is based on *IPR, finance knowledge development* and *academic goal* axes as in Fig. 1d. We label it *Contracted.* It emphasizes patented university-knowledge. In all the cases, university motivations are in finding funds to undertake research and increasing networks of research and industry partners*.* Industry motivations are split into getting support product development and finding new research directions and opportunities*.* This cluster is characterized by a focus on technologies to substitute for existing ones, high frequency of technological and cultural problems and outcomes that can be commercialized. These projects tend to be implemented under licensing agreements and as PhD research, involving frequent but formal communication between the partners.

In addition, the four types of projects identified seem to differ in terms of disciplinary diversity, the prior collaborative experience between the firm and the research team or the university department, and role of the partners in initiating the collaboration. The two most populated clusters, *Sponsored* and *Explorative,* reveal a large disciplinary diversity, especially the latter. The two least populated seem biased to specific disciplines: *Contracted* towards biology and chemical engineering and *Industrial* to applied physics and electrical/electronic engineering. Concerning prior collaborative experience, *Sponsored* projects represent to a large extent continuation projects. Only in two out of the 11 cases, the industrial partner had no prior experience with the specific researchers, but in these two cases, there was prior experience with the same university department. These projects have mostly been initiated to explore prior collaborative results or by part-time professors. On the other end, *Contracted* projects refer mainly to new collaborations, as in none of the four cases was there any type of prior experience between the university and the industry partners. Two cases were initiated by the industry and the other two by the university. In the *Explorative* and *Industrial* projects groups, only 2 and 1 projects, respectively, represent new collaborations. These two types of projects, especially in the *Explorative* group, present higher diversity in the role of the partners in initiating the collaboration. In the *Industrial* cluster, projects were somehow more often initiated by the industry and researcher mobility and in the *Explorative* cluster more often by university researchers, part-time professors, research mobility, and prior collaborations.

#### Examples of each type of university-industry projects

Here, we briefly describe four projects that can be considered typical Sponsored, Explorative, Industrial and Contracted university-industry collaborations.

One project representative of the *Sponsored Projects* cluster is the collaborative project that was undertaken as a follow-up development of a prior collaboration between a Dutch technical university and a manufacturer of transmission systems for automobiles. The collaborative project was sponsored by a government program to support environmentally-friendly research projects – especially through university-industry partnerships. The project involved three PhD researchers whose assigned task was to deliver a ‘proof-of-concept’. The project led to four patent applications, and to the creation of a spin-off company since the original partner company was not interested in diversifying and commercializing the invention, which still had to be developed beyond the embryonic stage.

One project representative of the *Explorative projects* cluster is the collaboration between the biomechanical Engineering department of a Dutch technical university, a Dutch academic hospital and a foreign firm. Two university researchers, one working also at the hospital, developed a theory for a novel method to measure flow in the coronary artery, which needed to be tested before being used clinically. Hence, the academics approached the private firm that had earlier developed relevant sensors. The partnership benefited from financial support from STW and from the private firm, which also provided equipment for the research performed at the university. The project was implemented by two PhD students, who conducted most of the research at the university, but there was intensive and quite informal interaction with the other two partners. The collaboration also allowed a firm researcher briefly to visit the university, and a university master student to do a Master thesis at the firm. The project resulted in an improved method based on the theory developed at the university, which came to be used in the partner hospital, as well as in a Belgian hospital.

A project representative of the *Industrial projects* cluster is the collaboration between a Dutch technical university and a governmental institute. An engineer from the governmental research institute approached some of his contacts at the university to get help on developing a new type of mirror, which was seen as a request from industry and experts in the technology area. The university researcher was interested on the knowledge development idea that the governmental research institute needed and a Master student was found to undertake the feasibility study. The master student performed most of the research at the governmental institute, and succeeded in developing a first concept of an extendible deformable mirror, which was patented. Given this success, the team decided to submit a fund request for a follow-up partnership, this time involving also another technical university and industrial firm.

A stereotypical project of the *Contracted project* cluster is exemplified by the collaboration between a foreign firm that learned about the university’s research at a conference and a Dutch technical university. The firm proposed a collaborative project (+2 years) to explore industrial applications of this work and tap into the university’s knowledge. The project was to be funded by the firm and a Dutch research institute. The firm patented the preliminary university results. Throughout the project, the university provided continuous feedback on research results and provided week-long training for some of firms’ researchers. The firm gave no feedback on its in-house testing and application after the first year. By the end of the first year of the project, other patents had been published, but the firm cancelled the collaborative project. The senior university researcher later learned that the firm had created a new R&D facility that allowed it to reproduce the university’s scientific research and proofs of concept and to proceed with development of a new product. Following acrimonious discussion in the university, the researchers found alternative funding sources and were allowed to continue their research in a four-year project, which resulted in publications and prestige for the university.

## Discussion

Results suggest that university and industry motivations are aligned in particular combinations. Some motivational spaces are in conflict, i.e., they describe how motivations of one kind (in university/industry) do not go well with motivations of another kind (in industry/university) and create trade-offs during the alignment process. We identified two trade-offs axes. One trade-off we labelled academic goals vs. advance industry research agenda. It is created by the juxtaposition of the university researchers’ interest in finding applications for their basic research results, which is in conflict and is difficult to be reconciled, with an industry motivation for accessing public sponsoring. If firms are motivated by accessing public sponsoring, university researchers seem more willing to join the project in order to maintain collaborative networks. The second trade-off concerns finance knowledge development vs. access technical support and results mainly from conflictual interests and goals of the industry. These results are in line with prior literature that has stressed the exploration-exploitation and the short-run versus long run trade-off in knowledge development activities, and also specifically between the industry and academic knowledge incentives structure (March 1991; Rosenberg and Nelson 1994; Nelson 2004; Gupta et al. 2006).

Three other motivational alignment spaces were identified. In one instance, the field of applied R&D, firm and university motivations were quite in parallel: university researchers were motivated by applicability of research, and firms were motivated by research opportunities in newly discovered technologies (with no immediate target market). This alignment space seems to match with the so-called Pasteur quadrant (Stokes 1997; Baba et al. 2009; Subramanian et al. 2013). Two other alignment spaces were identified associated with access to research financing and the presence of IPRs, and hence with the role of some institutional facilitators in encouraging motivational alignment. In one case, they relate mostly to university motivations to access future research opportunities (elaboration of patented basic knowledge); in the other they seem to be associated with the STW’s emphasis on IPRs.

Four types of projects —Sponsored, Explorative, Industrial and Contracted— recombine differently these motivational alignment spaces, observe specific organizational structure and the presence/absence of different institutional facilitators. In *Sponsored* projects, motivations associated with maintenance of research contacts (university) and access to research funds and new product development (industry) tend to be aligned in projects characterized by low levels of communication and formal organizational formats, often focusing on technologies to complement existing ones. *Sponsored* projects are strongly influenced by policy measures to fund collaborative research (in particular from the research funding agency STW), and, in several cases, they involve part-time professors.

Formal organizational and communication formats and commercialization of products are also the characteristic of *Contracted* projects. These projects are instead mainly characterized by being facilitated by existing patented university inventions, by university involvement motivated to open up (long-run) future research opportunities, and by industry involvement motivated by exploring new avenues for product development. *Contracted* projects tend to focus on developing technologies to substitute for existing ones and by high frequency of technological and cultural problems.

*Explorative* and *Industrial* projects rely much less on institutional facilitators. Some projects also benefit from policy sponsored initiatives, but they are less dependent on external incentives or funding. These projects have a much broader motivational basis in industry and university, and combine university-driven and business-driven motivations. When the university motivation is research applicability, projects tend to be characterized by intensive communication and focus on technologies to substitute for existing ones (i.e. in many *Explorative* and in some few *Industrial* projects). In this case, the degree of formalization of the communication and interaction between the partners, and the form of project implementation seem to vary according to the industry motivations to collaborate, and to the institutions that support the alignment of these motivations. PhD and Master students have been involved in most of the projects examined.

This evidence suggests that institutional facilitators are associated with collaborations that rely on formal implementation forms and on weak and relatively formal communication for problem-solving and knowledge transfer. This may be caused by the fact that external parties are involved in financing and managing the collaboration, the work packages of the partners involved are relatively sequential/ independent or the partners have few incentives to communicate frequently and informally. It might also be that institutional facilitators may be encouraging university-industry motivational alignment distant from Pasteur or Edison quadrants of scientific research proposed by Stokes (1997).^7^

Overall, our evidence suggests also that the presence of specific organizational formats tends to prevail in the presence of specific institutions (Nelson and Sampat, 2001). Hence, institutions and organizational formats may be complementary in creating resources and incentives in which specific axes of motivational alignment can occur. Nevertheless, occasionally organization structure may counteract or substitute institutional incentives (Nelson 1991). For example, the few *Explorative* projects that received public funding differed from the *Sponsored* projects. Despite benefitting from public funding, the parts managed to create an organizational and technological structure permitting these projects to address more exploratory research objectives and to achieve close to market outcomes. *Industrial* projects also permit the alignment of university motivation to maintain collaborative contacts and firms’ motivation of product development and/or solve technological problems. Still the organizational and technological structure of Industrial projects differ greatly from that of *Sponsored* in terms of length, technological scope, organizational structure and resources used (university and firm resources vs. public research funding).

## Conclusions and policy implications

The present study explored the complementarity/substitute relationship between organization and institutions by examining how the different axes of alignment of university and industry motivations for collaboration are integrated in projects with specific technological objectives and organizational structures, and benefit from the presence of certain institutions. We developed a conceptual framework and provided evidence based on 30 university-industry collaborations in the Netherlands, on how aligned motivations, institutional facilitators and project organizational formats go together.

Results suggest four main spaces of alignment of university and industry motivations (advance industry research agenda, finance knowledge development, access technical support and applied R&D) and two trade-off axes (academic goals vs. advance industry research agenda, and finance knowledge development vs. access technical support). Four different types of projects —*Sponsored, Explorative, Industrial and Contracted*— were identified. Each type of project is associated with certain technological objectives and organizational format of collaborative project, and with the presence of certain institutional facilitators. Only in few a cases did we observe similar motivational alignment in projects that occurred in the absence of certain institutional facilitators. However, the organizational formats of these collaborations were very different.

This study contributes to the three streams of the literature on which it builds. To organizational studies, which show that certain organizational structures are associated with certain outcomes and consequently to certain knowledge objectives (Gulati and Singh 1998; Osterloh and Frey 2000; Birkinshaw et al. 2002; Ranft and Lord 2002; Cummings and Kiesler 2007), this study contributes by proposing and providing evidence that organizational structure and technological objectives are set by researchers, exposed to specific institutional set ups, with specific motivations to relate and perform. To the economics of science and innovation, concerned with identifying the role of institutional facilitators on the setting-up and outcomes of the collaboration or on the role of researchers’ intrinsic and extrinsic motivations in the outcomes of university-industry collaborations (D'Este and Perkmann 2011; Lam 2011; Matt et al. 2012), this study contributes by suggesting that these analyses may need to try to disentangle the effects of the organizational structure and technological objective of the collaboration from those of the institutional facilitators and from researchers’ motivations. To the institutional approach, the study contributes by showing that specific organizational and technological structures tend to prevail in the presence of specific institutions, but also occasionally organization can substitute and/or counteract the institutional incentives.

Our results are very preliminary, hence further research using the university-industry empirical setting or other is needed to examine in which conditions organization can really counteract or substitute rather than complement the incentives provided by institutions or to examine whether causal relationships can be identified among these three dimensions (motivations, organization and institutions) or between specific types of projects and performance.

### Policy implications

Although exploratory, our analysis has some implications for policy. Projects that depend heavily on external funding (*Sponsored*) are characterized by a peculiar pattern of motivation biased towards advancing industry research agenda, rather than being driven by the research agendas of the university researchers. While we cannot establish how motivations influence the outcome of collaborative projects, our results suggest that university researchers’ “intrinsic” motivations (e.g., scientific curiosity) provide weak motivation in the sponsored projects group. Future research might provide more evidence on whether this may affect project outcomes.

Hence, it seems that there may be a low level of additionality of public sponsored research, especially if part-time professors are involved. In these cases, projects are often follow-up collaborations aimed at backing up technological development under existing technological frameworks. Dutch research foundations should take account of this and try to target a share of (and control) projects with high additionality (i.e. that develop knowledge in potentially interesting areas and explore industrial applicability of university results) and avoid a strong bias towards industry projects related to development of new complex products. If public research funding is encouraging university-industry alignment of distant from the academic goal, in the long run this may be reduce the value of collaboration, hence being detrimental to both parties (Lam 2011).

Furthermore, the analysis provides some insights into the role of IPRs in knowledge transfer, an important policy topic. Projects (*Contracted*) that focus on developing university-patented knowledge seem more likely to be the basis for new collaborative contacts and the development of substitute technologies that usually succeed in developing advanced prototypes and lead to plans for product commercialization. At the same time, these largely industry-financed projects tend to experience managerial problems and several coordination conflicts resulting from the different research culture of the parts, which waste time that could be spent on research or education. Our exploratory results are in line with prior literature that showed that licensing activities may not have a negative effect on research productivity (publications and citations) of academics, but it can decrease ability to get government grants (Thursbyn and Thursby 2011), possibly reflecting their decrease ability to target funds for somehow more blue sky research. We provide evidence, as Bessen and Meurer (2008) did earlier, of the double-edge of academic patenting as policy instrument to facilitate knowledge development and transfer.

Finally, our results suggest that policy should recognize the power of collaborative university-industry Masters and PhD theses tools to promote university-industry interaction. In particular, Master theses facilitate the motivational alignment for developing pre-feasibility studies for industry application. However, as some authors have stressed, graduate student traffic is often exploited by academics to strengthen collaboration with firms, and may have consequences for the culture of science and research (Slaughter et al. 2002).

### Research limitations and future research

Our study has some limitations that open up avenues for future research. First, in order to extract detailed information on collaborations, our analysis used unique project level data and, necessarily, relied on a small sample of observations. This restricts the type of statistical analysis possible and makes our results exploratory. Further research is needed on larger samples using additional methods of enquiry and analysis. A larger sample would also permit us to ask whether other specific characteristics of industry and university partners play a role in explaining the space of motivational alignment achieved and the characteristics of the project.

Second, we relied on cases of university-industry collaboration in the Netherlands. Some characteristics of the motivational spaces and their relationships with specific collaboration formats might be specific to the Dutch institutional environment in relation to university regulations, academic career progression, science policies, and the functions of research councils. Future research could examine whether these specific findings can be generalized to other countries, given cross-country differences in institutions and careers.

## Footnotes

1 Among our 30 collaborative projects, we identify three levels of knowledge transfer: transfer (without recognition of value nor exploitation by the firm), knowledge absorbed (firm recognized the value of the knowledge but did not use it), knowledge exploited (in further research, further product development, process improvements, or commercialization of new products).

2 In two of the 30 collaborative projects, the scientific or technological objectives (i.e. those defined at the start of the project) were not realized; in four cases the outcomes exceeded expectations; in 17 cases, projects led to plans for commercialization of new products. However, universities evaluated 26 projects as fully positive, while firms were more critical and reported the same level of satisfaction in only 21 cases.

3 The Technology Foundation STW, which was created in 1981, was set up to stimulate technical scientific research and its utilization, and to encourage public-private knowledge transfer by allocating funds to collaborative industry-university research. Its main goal is to bring together public and private organizations through cooperative research arrangements, which may result in practical applicable results of patentable quality.

4 The characteristics of the local industry and the university disciplinary specialization permitted the development of specific relationships, such as Eindhoven university with Philips. The universities involved in our collaborative projects specialized in different applied disciplinary fields. They differ in their specialization in knowledge development for “science-based” and “development-based” technologies (Nelson and Winter 1982). Note that academic careers and wages are largely fixed nationally, either by central government policy or by collective wage bargaining. Similarly, the rules for academic IPRs and TTO activities are mostly defined by central government policy. However, some experimentation with regard to TTOs and IPRs is possible at university level. Overall, the institutional rules and practices of each university do not seem to influence radically the way collaboration and technology transfer occurs in Dutch universities. In seven cases, more than one university or a university and a research institute were involved in the design and often the performance of the development project.

5 In projects with three partners, all partners were interviewed; in the cases (5) of larger collaborations only the partners involved in the development of the piece of knowledge were interviewed.

6 The university motivations revealed by the university partner fall into distinct codes already identified in the literature, and hence were retained as such. The motivations revealed by the industry partners, instead, had to be reworked. First, we dropped the motivation “Access to IPR” once it coincided with variable “Previous university patented knowledge”. Second, in two thirds of the cases the industry partners reported two almost fully coinciding motivations: “Need to develop new knowledge” and “Need university skills, expertise and facilities”. These two codes were merged and re-coded using information on the reasons behind the need for that new knowledge and university skills: “New product development” and “technological problem”. Finally, motivations “Access to public funding” “Research opportunity” were kept as revealed by the interviewees.

7 While none of our four clusters portray with accuracy any of the Stokes (1997) quadrants, some relationships can be forced. *Explorative* projects might be understood as reflecting the Pasteur quadrant, i.e. involving basic research (but, in our cases, also often a lot of engineering work) aimed to solve practical problems. *Industrial* projects could be forced into the Edison quadrant, portraying applied research targeting practical problems. Instead, *Sponsored* and *Contracted* groups seem a mix between the Edison and the unlabelled quadrants (i.e. research targeting questions emerged from previous research results, i.e. reflect the random walk nature of science). In Sponsored projects, research problems often emerge in past collaborations, while in Contracted projects, research problem follows up university patented research driven by curiosity to understand natural phenomena.
